# Awareness of antibiotic resistance and antibiotic prescribing in UTI treatment: A qualitative study among primary care physicians in Sweden

**DOI:** 10.3109/02813432.2012.751695

**Published:** 2013-03

**Authors:** Ingeborg Björkman, Johanna Berg, Nina Viberg, Cecilia Stålsby Lundborg

**Affiliations:** ^1^Department of Public Health and Caring Sciences, Health Service Research, Uppsala University, Uppsala, Sweden; ^2^Department of Public Health Sciences, Division of Global Health (IHCAR), Karolinska Institutet, Stockholm, Sweden

**Keywords:** Antibiotics, bacterial resistance, general practice, general practitioners, interviews, urinary tract infection, Sweden, views

## Abstract

*Objectives.* To improve education and information for general practitioners in relation to rational antibiotic prescribing for urinary tract infection (UTI), it is important to be aware of GPs’ views of resistance and how it influences their choice of UTI treatment. The aim of this study was to explore variations in views of resistance and UTI treatment decisions among general practitioners (GPs) in a county in Sweden. *Design.* Qualitative, semi-structured interviews were analysed with a phenomenographic approach and content analysis. *Setting.* Primary care in Kronoberg, a county in southern Sweden. *Subjects.* A purposeful sample of 20 GPs from 15 of 25 health centres in the county. *Main outcome measures.* The variation of perceptions of antibiotic resistance in UTI treatment. How UTIs were treated according to the GPs. *Results.* Three different ways of viewing resistance in UTI treatment were identified. These were: (A) No problem, I have never seen resistance, (B) The problem is bigger somewhere else, and (C) The development of antibiotic resistance is serious and we must be careful. Moreover, GPs’ perceptions of antibiotic resistance were mirrored in how they reported their treatment of UTIs in practice. *Conclusion.* There was a hierarchal scale of how GPs viewed resistance as an issue in UTI treatment. Only GPs who expressed concerns about resistance followed prescribing guidelines completely. This offers valuable insights into the planning and most likely the outcome of awareness or educational activities aimed at changed antibiotic prescribing behaviour.

- The level of antibiotic use correlates to the level of antibiotic resistance; therefore infection treatment guidelines promote rational antibiotic prescribing.
- A perception that antibiotic resistance must be considered was related to adherence to all aspects of UTI treatment guidelines.
- A perception that enough was already done to contain resistance was related to non-adherence to all aspects of UTI treatment guidelines.

## Introduction

Antibiotic resistance is increasingly challenging health care [1]. Irrational use of antibiotics is considered to contribute unnecessarily to increasing the rate of resistance [2]. Studies show that it is possible to decrease antibiotic prescribing [3,4]. However, more efforts are needed to implement changes [5–7]. In Swedish primary care uncomplicated urinary tract infection (UTI) is one of the most common diagnoses for which antibiotics are prescribed [8,9]. UTI treatment is usually empirical. In Sweden continual alternative use of first-line drugs is recommended for a duration of 3–5 days, and flouroquinolones are not recommended as first-line drugs, as they are needed in more complicated infections [9]. Today the recommended first-line drugs are pivmecillinam and nitrofurantoin [9]. When this study was conducted in 2004 trimethoprim and cephadroxil were also recommended as first-line drugs [10]. Extended durations and unnecessary prescribing of flouroquinolones were reported for UTI treatment [11].

The most common pathogen involved in UTI is Escherichia coli. From 1983 to 2001 the level of E. coli resistance to trimethoprim among outpatients in Sweden increased from zero to 12% [12,13], and the level has continued to increase; it was 20.3% in 2009 [14]. In 2004 most studies performed on UTI treatment were focusing on quantifiable aspects [8,10,11], and less was known of how resistance and its influence in the choice of UTI treatment were viewed by the prescribers. An opportunity to explore this issue was at hand along with the decision to reduce trimethoprim prescribing in a county in Sweden in 2004 [15]. The aim of this study was thus to explore variation in views of resistance and UTI treatment decisions among GPs in the county.

## Material and methods

Based on the aim of this study two qualitative research methods were chosen [16]. These were the phenomenography approach to identify and describe different ways of understanding antibiotic resistance [17], and content analysis to capture views of UTI treatment decisions [18].

### Sampling procedure

The study included 20 GPs in Kronoberg, a county in southern Sweden with a population of 178 000 inhabitants and about 80 GPs. Purposive sampling was used to achieve variations in age, sex, and geography. The GPs were contacted by one of the authors (JB) and asked to participate. Seven of the selected GPs initially declined and a sufficient number of subjects was reached after further contact with GPs fulfilling the inclusion criteria. The GPs (Table I) were working at 15 of 25 health centres, in six of eight municipalities in the county.

**Table I.** Gender, age, and geographical distribution of the GPs interviewed.

Characteristics			
Gender	Women	Men	Total
Age			
31–40	2	1	3
41–50	4	5	9
51–60	2	5	7
61–70	–	1	1
Geographical location			
Rural area	4	5	9
Urban area	4	7	11
Total	8	12	20

### Data collection

Data were collected by face-to-face interviews following a pre-tested, semi-structured interview guide (Table II), containing open-ended initial questions. Probing questions enabled the interviewer to follow up on the answers. The interviews were performed by one of the authors (JB) during October and November 2004, at the GP's workplace, at the Research and Development Centre in Växjö, or in the GPs’ homes. The interviews lasted 15–40 minutes, were tape-recorded, and were transcribed verbatim.

**Table II.** Interview guide.

Area	Introductory questions	Probing questions
Introduction	Could you please think of a patient with uncomplicated urinary tract infection and describe this patient?	Typical patient? Do you often meet patients like that? What happens when patients like that comes to you? Symptoms? Diagnosis?
Treatment	How did you decide on the treatment for this patient?	Symptoms/urine tests? Direct/delayed treatment? Patient attitudes? Choice of drug? How frequently do you use that drug? Continual alternative use of first-line drugs? Treatment duration?
Effect of treatment	How do you usually experience the effect of the treatment?	Recurrent symptoms? What happens if the symptoms return? Similar diagnosis? Have you seen treatment failure associated with resistance?
Resistance	What are your thoughts on resistance in general?	Influence on daily work? The microbiological laboratory? Update? Influence of antibiotic use? How can you curb the trend?
Is there anything else you would like to say?		

### Data analysis

The phenomenographic analysis was first performed by two of the authors separately (JB and IB). The analysis followed a sequence of steps described by Alexandersson [19]:

(1) becoming familiar with the data and gaining an overall impression;

(2) noting similarities and differences in the statements;

(3) determining descriptive categories of conceptions; and

(4) examining the underlying structure of the system of categorization.

The findings of the two analysts were compared and the final categories of description were established in a discussion between the analysts and the two last authors (NV and CSL), who were also familiar with the material.

During the additional content analysis [18], performed by JB and IB, statements concerning treatment of UTI were gathered and coded. Then the codes were summarized to make up a description of each GP's practice of UTI treatment. The relationships between described practices and identified perceptions were examined. This part of the analysis was discussed among the researchers and the final result was approved by all authors.

The study was approved by the Ethics Committee in Linköping (Dnr 03-04). Informed consent was obtained from all participants.

## Results

### Perceptions of resistance in UTI treatment

Three different, mutually exclusive ways of viewing resistance in UTI treatment were identified in the phenomenographic analysis. They were: (A) No problem, I have never seen resistance; (B) The problem is bigger somewhere else; and (C) The development of antibiotic resistance is serious and we must be careful. In Table III the three categories are described and complemented with illustrative quotations, chosen so as to reflect the view of resistance in each category. In general the physicians said that UTI was easy to diagnose and to manage.

**Table III.** The three identified perceptions.

Category of description	Quotation
A. No problem, have never seen: Resistance was not seen as a problem in the everyday treatment of UTI. The GPs thought that practical problems seldom appear or that they had not experienced treatment failures due to resistance. In this perception, an antibiotic resistance problem at the global level was not considered nor were future consequences.	As for me, I prescribed for example trimethoprim to almost 90% of my patients, a trimethoprim preparation of short duration, five days. It is enough because it is effective…. No, I have never experienced that [patients cannot be treated by an antibiotic due to resistance]. This is not how it used to be. (Dr 9)
B. The problem is bigger somewhere else: In this perception there was an awareness of the existence of the antibiotic resistance problem. However, this had no or minor impact on the GP's own practice. Some GPs said they must sometimes change antibiotics due to resistance but this was not really a problem. The notion was that the development in Sweden was less urgent than in other countries, or there was a feeling that great efforts are already being made to curb the trend. It was also expressed that maybe the resistance problem was worse in large cities in Sweden but not here in the countryside, or in hospital care but not here in primary care.	The cases where they don't get well, that is resistance. But, then you have to switch drugs and you can usually do that. There are other drugs to choose. You seldom feel totally helpless…. As I see it in Sweden about the urinary tract infections, it doesn't feel like a big problem for me right now…. If the trimethoprim susceptibility or the resistance rises a bit, it still doesn't feel like a big problem. (Dr 14)
C. The development of AR is serious and we must be careful: Also in this perception was the notion that the problem of antibiotic resistance was worse in other countries and in hospital care and management of UTI was generally not seen as a problem. However, the GPs in this category reflected in broader terms on future consequences and expressed concerns about the increasing resistance. It was said that doctors must take the resistance problem seriously and be careful in the prescribing of antibiotics in order to contain the development of antibiotic resistance.	Even if you see resistance in vitro, in the test tube, it may still have been effective in vivo, in real life…. But it's worrying that resistance to certain drugs is increasing more and more…. It's also rather worrying that no new antibiotics are being developed … and so, so we have to be careful with the ones we have. (Dr 8)

### UTI treatment in practice and correlation to perception

In the content analysis, two elements of UTI treatment were examined. They included: (1) the choice of antibiotic and (2) the treatment duration. This part analysed the GPs’ thoughts about UTI treatment in practice and how their practice (according to themselves) correlated to the identified perceptions A–C.

### 1. Choice of antibiotic

The GPs mainly discussed issues concerning the recommendation to alternate among first-line drugs. Many of them thought that this should be applied in the treatment of a single patient to avoid recurrent infections due to resistance. Some GPs were critical and stated that every infection should be seen as a new infection and that a previously effective prescribed drug could be used again. Instead of alternating, a specific drug was commonly perceived as safe in most situations and hence used in a habitual manner. Lack of records, fear of side effects, patient demand, and costs were mentioned as reasons for the repeated use of a specific drug.

All GPs who expressed perception C reported that they practised continual alternative use of first-line drugs both in individual patients and in the population. These GPs said furthermore that they were careful in the use of flouroquinolones, whereas some of the GPs expressing perceptions A and B reported sporadic use of flouroquinolones as first-line drugs.

### 2. Treatment duration

Many of the GPs expressed reluctance to follow recommendations for shorter treatment durations. A very few questioned the effectiveness, whereas others found it difficult to change their previous ways of thinking. All GPs who expressed perception C reported that they followed the guidelines and used shorter courses of antibiotics of 3–5 days.

## Discussion

### Principal findings

Three separate views of resistance in relation to UTI treatment were identified. The views varied from (A) not seeing resistance as a problem at all in the treatment of UTI, to (B) explaining that the problem does not exist here but somewhere else, in other countries, big cities, or in hospitals, to (C) the notion that there is no real problem today but we must take this issue seriously and be careful.

Only GPs who expressed perception C said they practised UTI treatment according to the stated guidelines in all aspects (continual alternative use of first-line drugs, and short treatment duration).

GPs who expressed perceptions A or B also reported that they followed guidelines for UTI treatment but did not do so in all aspects. For instance, they said they practised the alternative use of drugs but only at the individual level and often for seven days. Or short treatment was used but without alternating, or alternating was used but with flouroquinolones as one of the drugs. Many of these GPs explained that they were aware of all recommendations and knew they must change their practice. However, there was also a common belief that the precautions taken were good enough.

### Strengths and weaknesses of the study

The analysis of perceptions followed experience indicating that 20 participants are enough in an interview study aiming at exploring different ways of viewing a phenomenon [20–22]. To increase trustworthiness the analysis was first done separately by two of the authors, one with long experience of phenomenographic analysis (IB), and in a second step categories were established in a discussion between all of the authors.

A weakness of the results of the study is that the interviews were held in 2004 and the awareness of antibiotic resistance may have developed since then. However, the problem of antibiotic resistance is not less today; instead it is more timely than ever. New strategies in the promotion against antibiotic resistance are still needed. We believe our conclusions are relevant and valuable.

The second part of the analysis examined the GPs’ perception of resistance with the GPs’ reported decisions in the treatment of UTI. A weakness is that we have not studied how the GPs practise in real life. We only know this from what the GPs explained during the interviews. However, the questions were constructed to help the GP to think about a real patient situation and make the collected interview material as true as possible.

### Comparison with other studies

Deviations from recommended treatment have been reported, such as the use of second-line drugs and extended treatment durations [23,24]. Prescribing has been described as done in a habitual manner [3,23–25], based on the trust of broad-spectrum antibiotics as being more effective [26], and being influenced by the structure of the healthcare system [25]. In primary care the patient–physician relationship seems to influence how antibiotics are prescribed [27,28]. However, in hospital care other factors aside from the relation to the patient were important when physicians decided how to manage infections [29].

Some studies explore whether physicians consider antibiotic resistance or not when infections are managed. GPs have reported concerns about antibiotic resistance but state that they must consider many factors in deciding whether or not to prescribe an antibiotic, and often patient issues are regarded as being more important than the issue of antibiotic resistance [30,31]. Physicians who stressed patient issues were less likely to use many resources (urine culture, microscopic urinalysis, follow-up visits and tests, and prolonged antibiotic treatment) and more likely to recommend treatment over the telephone [31]. However, it has been demonstrated that symptoms of UTI are not conclusive for the diagnosis of uncomplicated UTI [32]. In our study GPs were aware of antibiotic resistance but only a few said that this factor must be specifically considered in the treatment of UTI. The issue of antibiotic resistance may also be thought of as a national problem that does not concern the physician's own practice [33], a concept that was also seen among participants in our study. When the physicians considered antibiotic resistance as an important factor, they did follow recommended regimens more often, according to their own reports [28,29].

### Meaning of the study

Our findings suggest a relationship between compliance with treatment guidelines for UTI and the perception that antibiotic resistance is a major problem. It seems that when the GP has not adopted this perception he or she is satisfied to follow parts of the recommendations and believe this is good enough. To successfully promote cautious antibiotic prescribing it is probably of importance that the GPs become fully aware of possible threats from developing resistance, with regard to both their own practice and long-term circumstances.

### Unanswered questions and future research

The findings in this study contribute to the understanding of physicians’ ways of thinking about antibiotic resistance and antibiotic prescribing. However, qualitative studies are used to develop hypotheses and our findings can preferably be verified in further studies with a quantitative design. Other factors likely to influence the GPs’ prescribing behaviour, such as patient demand or the GPs’ personalities, were not evaluated. Further research in this area is desirable in order to provide insights into the importance of views on resistance and the changes in prescribing behaviour.
